# Association of Formal and Informal Social Support With Health-Related Quality of Life Among Chinese Rural Elders

**DOI:** 10.3390/ijerph17041351

**Published:** 2020-02-19

**Authors:** Shan Lu, Yupan Wu, Zongfu Mao, Xiaohui Liang

**Affiliations:** 1 School of Health Sciences, Wuhan University, Wuhan 430071, China; 2 Global Health Institute, Wuhan University, Wuhan 430072, China

**Keywords:** social support, health-related quality of life, population aging, Chinese rural elders

## Abstract

*Objectives*: To explore the association of formal and informal social support with health-related quality of life (HRQOL) among Chinese rural elders and further investigate the influence of quantity and quality of social support on their HRQOL. *Methods*: The sample of 4189 Chinese rural elders over 60 years old was acquired from the 2015 China Health and Retirement Longitudinal Study (CHARLS). The HRQOL was evaluated by EQ-5D-3L questionnaire. The social support assessment was mainly based on the social support rating scale (SSRS), and Tobit regression analysis was used to explore the impact of social support on HRQOL. *Results*: The average EQ-5D index score (0 to 1) of the Chinese rural elders was 0.78 ± 0.16. Participants who were male or with better education were found to have higher scores. Those elders living alone, suffering from chronic diseases or disabled acquired lower scores. As for formal social support, higher medical or pension insurance and more social activities statistically significantly possessed higher scores. As for informal social support, higher number of offspring had a significant association with lower scores, while more contact with children and financial support from family were shown to be statistically significantly associated with higher scores after controlling for sociodemographic characteristics. The quality of social support is more important than its quantity. An interesting finding was that the EQ-5D index scores did not support the Chinese traditional belief that ‘the more children, the more blessings’. *Conclusions*: The EQ-5D index scores of the rural elders in China is above the median level based on the scores of EQ-5D. Social support is significantly associated with elderly peoples’ quality of life. The results would be significant for accurately improving the life quality of Chinese rural elders from the perspective of social support.

## 1. Introduction

As important research relevant to the aging population, health-related quality of life (HRQOL) has been attracting increasing attention from domestic and foreign scholars in recent years [1,2,3,4]. The World Health Organization (WHO) defined HRQOL as an ‘individual’s perception of their position in life in the context of the culture and value system in which they live and in relation to their goals, expectations, standards and concerns’ [5]. Previous studies have shown that HRQOL can be affected by sociodemographic characteristics, socioeconomic status, health care policies and dangerous behaviors, etc. [6,7,8]. In recent years, an increasing number of studies have been conducted to explore the relationship between social support and HRQOL, demonstrating that higher social support could effectively relieve the pressure on individuals and increase their subjective wellbeing [9,10,11].

China is one of the leading countries with respect to the speed of its population aging. According to statistics, more than 241 million people were over 60 years old in 2017 in China, and this number is predicted to reach about 487 million by 2050, accounting for 34.9% of the total population [12]. Compared with the situation in other countries, China’s population aging faces more serious problems with respect to aspects such as large scale, rapid growth, regional imbalances, inversion of urban and rural areas, and aging under poor conditions [13,14]. The manner in which quality of live can be continuously improved is an important social issue that the Chinese government and the whole society ought to be highly invested in, both at present and in the future.

In the 1970s, social support emerged as a technical term of psychiatric literature [15]. Since then, social support has been widely used in psychiatry, medicine, sociology, psychology and other disciplines [16]. Now, social support has become a multi-dimensional topic, along with the enrichment of the implications of social support, and researchers in different fields have defined social support differently according to their research purposes and backgrounds. So far, there is no unified definition of social support, and scholars have classified different kinds of social support based on their own knowledge structure, research purposes, and emphases. 

In general, social support can be classified with respect to aspects of its function and structure. In terms of the function of social support, Pryor used a dichotomy to divide socially supported functions into instrumental and emotional support [17]. LaRocco identified four types of social support: evaluation support, emotional support, instrumental support, and information support [18]. Cohen and Wills further summarized the previous research results and divided social support into four major functions: emotional support, information support, friendship support, and instrumental support [19]. According to the provider of social support, social support can be structurally divided into formal and informal social support [20]. Formal social support refers to the material and spiritual assistance provided by formal organizations in accordance with relevant policies or laws. This kind of assistance has the characteristics of regularity and stability, which shows the support relationship between organizations and individuals. Informal social support refers to the help provided by informal organizations or individuals, which is normally characterized by uncertainty. The formal social support provides include government, institution, unit, community and other formal social organizations, while informal social support provides include family members, relatives, neighbors and friends.

Furthermore, many studies have explored the relationship between social support and individuals’ physical health, mental health and subjective wellbeing. Some research has suggested that social support has a positive effect on the health of the elderly in both the developed and developing countries. For example, Cobb and Sidney considered social support to be a moderator of the life stress, helping people improve health status and social functions [15]. A survey of older persons in rural communities in Malaysia found that good social support could reduce the incidence of depression and anxiety in the elderly [21]. In one study across China, India and Latin America found that low levels of social support could increase the risk of death among the elderly [22]. Another study found that social support from their children played an important role in the elderly’s mental health, and that it was a positive factor. Friends, neighbors and social participation were also good for the elderly’s mental health [23]. 

Previous research has suggested that the quantity and quality of both formal and informal social support affects elderly people’s life satisfaction and subjective wellbeing. Tao and Shen reported that formal social support, such as medical insurance and pension insurance, produced a ‘buffer model’ effect on the physical and mental health of the rural elders [20]. Vandervoort and Debra studied the relationship between informal social support and mental health, and distinguished the impact of the quantity and quality of social support on physical and mental health, and the results suggested that the quality of social relationships had greater impact on mental and physical health than the quantity [24]. Guo and Liu studied the effect of core family members to the elderly life satisfaction, and the results showed that with support from families, the elderly had the highest satisfaction, and the number of children had a positive influence on quality of life in the elderly [25]. Some studies found that with more children, parents gained more financial support [26,27]. However, some studies found that more children would produce more conflicts and economic contradictions, leading to negative life satisfaction and subjective wellbeing, indicating that life satisfaction for elderly people was inversely proportional to the number of children [28].

In addition, many studies have focused on the relationship between social support and HRQOL [29,30,31,32,33,34]. However, most of these studies have concentrated on the impact of informal social support (from spouses or relatives) on the urban elderly’s HRQOL [32,33,34]. Yet, in China, 65% of the elderly live in rural areas. Compared with the elderly in urban areas, the elderly in rural areas fell behind in terms of formal social support such as social security [35]. Considering that many rural seniors are often left behind and need support from home- and community-based services, the role played by formal social support in promoting their functional autonomy and improving their quality of life presents a large gap in present research. In addition, the sample of previous studies mainly comes from specific local regions, failing to capture nationwide population preferences [36]. Finally, previous studies have typically used components of HRQOL as intermediary variables to explore the relationship between social support and HRQOL, without fully taking advantage of the effects of the index [37].

In this work, the objective is to explore the association between formal and informal social support and health-related quality of life (HRQOL) among the Chinese rural elderly in order to confirm whether social support plays an important role in the life quality of the Chinese elderly, and to further investigate the influence of the quantity and quality of social support on their HRQOL.

## 2. Materials and Methods 

### 2.1. Design, Setting and Sample 

The data of the study were derived from the survey of the 2015 China Health and Retirement Longitudinal Study (CHARLS), which was carried by the National Development Research Institute of Peking University. CHARLS aimed to collect a high-quality, nationwide, and representative sample of Chinese residents aged 45 or older to serve the needs of scientific research. The baseline national wave of CHARLS was fielded in 2011, and included about 10,000 households and 17,500 individuals from 150 cities/districts and 450 villages/resident communities. By the national follow-up investigation in 2015, its sample covered 23,000 participants from a total of 12,400 families.

After obtaining approval from CHARLS, while assuring the presence of all the key data, we included the data of the elders who (1) had a rural household registration in China, (2) were born before December 31, 1955, according to their ID cards, and where (3) within-household clustering was present [38]. In addition, the study only retained the samples of the primarily interviewed participants to ensure the independence of the samples. Finally, the data of 4189 elderly people were considered to be consistent with the inclusion criteria, and were selected for further studies.

### 2.2. Measurements

#### 2.2.1. Health-Related Quality of Life

The EQ-5D-3L was utilized in this study to measure the HRQOL of the Chinese rural elderly. This method evaluated five dimensions of HRQOL: Mobility, Self-Care, Daily Life Activities, Pain and Discomfort, and Anxiety and Depression. Each dimension was divided into three levels: no problem, moderate problems, and severe problems. In the study, the Cronbach’s α of the scale was set at 0.89. A utility integral system conversion table [39], which converts respondents’ choices in the three dimensions of quality of life into EQ-5D index scores, was adopted to evaluate the overall HRQOL of the elderly. The score varies from 0 to 1, where higher scores (closer to 1) stand for better quality of life.

#### 2.2.2. Social Support

In China, the social support system for the rural elderly consists of formal social support, provided mainly by the government and communities, and informal social support, provided by family members and friends. To be specific, the government mainly provides a social welfare system, including basic medical insurance and pension insurance; communities offer social network support; and family provides emotional support and instrumental support.

With respect to the CHARLS questionnaire, the social support rating scale (SSRS) was used as a reference for evaluating social support. In this study, we developed and validated a new scale based on the SSRS and the CHARLS variables. The SSRS was developed by Xiao, and is one of most commonly used instruments for measuring social support in China [40]. 

In this study, the quantity of formal social support was defined by the number of social activities, basic medical insurance and social pension insurance. The quality of formal social support was evaluated based on the frequency of participation in social activities (social network support) and the monthly income provided by social pension insurance. The quantity of informal social support was defined with reference to marital status, the number of children, and the number of grandchildren. The quality of informal social support consisted of emotional support and instrumental support. Among these, emotional support includes: the frequency of meeting with their most-visited children, the frequency of contact with children who do not live with them (including telephone, mail, etc.) and whether they were taking care of their grandchildren. Instrumental support included whether they were receiving any financial support from parents, children, and relatives.

### 2.3. Control Variables

To minimize the influence of confounding factors, age, gender, education level, marital status, living style, number of chronic diseases and disability were considered as control variables. Among these factors, marital status was described as being either married (including living together) or other (reference group), which included single, widowed and divorced. Education level was categorized as illiterate (reference group), primary school, middle school, and high school or above. Living style was divided into living alone (reference group) and living with others. Because chronic diseases and disabilities are frequently encountered by rural residents, they were also used as key control variables. Most of the data were converted to categorical variables (including binomial and multivariate variables) to facilitate analysis.

### 2.4. Statistical Analysis

Stata (version 14.0, StataCorp, College Station, Texas, USA) was used for data analysis, and statistical significance was set at *p* < 0.05. The continuous variable, such as EQ-5D index scores, was described as ¯x±s, and categorical variables such as demographic characteristics were expressed as the adoption rate or composition ratio (%). The Wilcoxon rank-sum test, Kruskal-Wallis test and Spearman rank correlation analysis were used to examine the differences between groups in these variables, and the test statistics were expressed as Z, H and rs, respectively.

**Figure 1:**
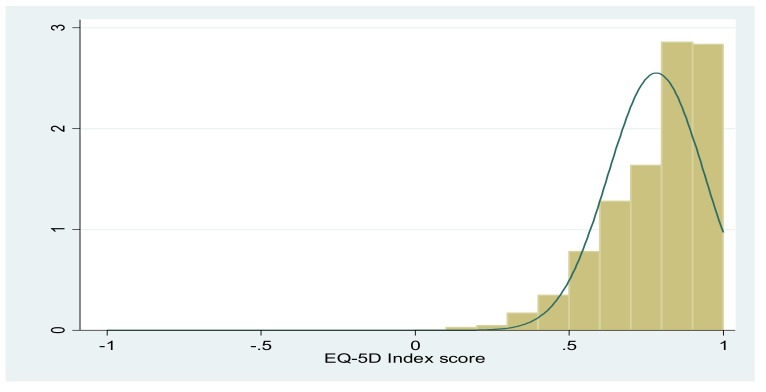
Distribution of the EQ-5D index scores by density.

Since the EQ-5D index score was a censored variable, for example, the majority of respondents had utility values of 1 in the study, and utility values less than 1 were continuous (Figure 1), the Tobit regression model was considered to be suitable for bounded or censored data. In the models, EQ-5D index scores, age and income were treated as continuous variables, and other variables were treated as categorical variables and were modelled using dummy variables (including missing dummy variables).

## 3. Results

### 3.1. Characteristics of the Participants 

The data for a total of 4189 elderly people were selected from CHARLS. Since the data was acquired from open resources, the study received a waiver from the IRB review of the university. 58.49% of the respondents were males, 71.52% were married, 25.14% were uneducated, and 64.88% were living alone. 3121 (74.50%) of the participants suffered from at least one chronic disease, and 1224 (29.22%) people had different degrees of disability (Table 1).

### 3.2. The Relationships Between Social Support and HRQOL

Figure 1 shows the frequency density of the EQ-5D index scores among all participants. The X-axis is the EQ-5D index score, while the Y-axis represents the density of the scores. The definition of density is frequency divided by group distance, and the area of the rectangle represents the frequency for the group. As illustrated in Figure 1, the frequency distribution of the scores was highly and positively skewed. The frequency analysis shows that most of the scores ranged from 0.8 to 1.

Table 1 indicated the correlation between EQ-5D index scores and sociodemographic characteristic variables. The mean EQ-5D index scores of the participants was 0.78 (SD = 0.16). The EQ-5D index scores were negatively correlated with age. While those who were male, married, or better educated had higher scores, those participants with at least one chronic disease or who were disabled had lower scores. 

Table 2 presents the correlation between EQ-5D index scores and formal social support. The EQ-5D index scores were significantly influenced by the number of social activities and number of types of medical insurance. The correlation indicates that the participants who had medical and pension insurance or who often participated in social activities had higher scores. Additionally, the EQ-5D index scores increased with the amount of pension insurance received monthly. 

Table 3 indicates the association between EQ-5D index scores and informal social support. The differences in the EQ-5D index scores between ‘whether having spouse’, ‘number of children’ and ‘number of grandchildren’ were statistically significant. Those among the elderly who reported that they ‘were often in contact with their children who they did not live together with’ had higher scores than those who ‘were never in contact with their children’. Those who ‘take care of grandchildren’ often had higher scores. In addition, high ‘financial support from their family’ indicated higher EQ-5D index scores.

### 3.3. Tobit Regression Analysis of Social Support and HRQOL

Table 4 shows the association between EQ-5D index scores and social support after adjustment for the evaluated demographic, socioeconomic and health status variables, with coefficients and standard error, as indicated using the Tobit model. 

In Model 1, EQ-5D index scores decreased as age increased. Males and higher educated participants tended to have higher EQ-5D index scores. Respondents who were married or disabled had lower scores. In addition, the EQ-5D index scores among those with at least one chronic disease were significantly lower than those who did not have any chronic diseases. In Model 2, the quantity of formal social support was included. The results indicated that the amount of medical insurance (β = −0.022, *p* < 0.05) and the urban and rural residents’ pension insurance (β = −0.034, *p* < 0.05) were significantly associated with lower EQ-5D index scores. Contrarily, EQ-5D index scores were higher among those who had a greater number of social activities (β = 0.016, p < 0.001) and a greater amount of urban and rural resident medical insurance (β = 0.032, *p* < 0.05) and government medical insurance (β = 0.047, *p* < 0.05). In Model 3, the quality of formal social support was included. Those among the elderly who often participated in social activities (β = 0.017, *p* < 0.05) had higher scores. The greater the amount of pension insurance members of the elderly received each month (ln) (β = 0.023, *p* < 0.001), the higher their EQ-5D index scores. In Model 4, the quantity of informal social support was included. The results indicated that only the number of children (β = −0.005, *p* < 0.01) had a significant association with lower EQ-5D index scores. In Model 5, the quality of informal social support was included. Participants that were in constant contact with children (β = 0.012, *p* < 0.05) and who received more financial support from family each month (β = 0.011, *p* < 0.001) had higher scores. 

The sociodemographic characteristics of the participants that were negatively correlated with the EQ-5D index scores in the fully adjusted model included older age, greater number of chronic diseases, and more disabilities. As for higher level of education, it was positively correlated with EQ-5D index scores. Additionally, the number of social activities (β = 0.009, *p* < 0.05), the frequency of social activities (β = 0.016, *p* < 0.05), the amount of pension insurance members of the elderly received each month (β = 0.024, *p* < 0.001), and the number of children (β = −0.009, *p* < 0.001) differed very little from those identified in the other models, which suggested that the association between EQ-5D index scores and social support was independent and stable. However, the association between HRQOL and marital status, and the amount of medical insurance, all types of medical insurance and pension insurance was not statistically significant after controlling for sociodemographic characteristics.

## 4. Discussion

In this study, the association between HRQOL and formal and informal social support was evaluated in a specific population: the rural elderly in China. The elderly are often considered as a vulnerable group, and their quality of life is generally assumed to be relatively low. Therefore, in the rural areas of China, the traditional belief that more children should be raised in order to protect the aged is very popular. Many among the rural elderly hope to have more offspring, especially more sons. This study focused on the association between quality of life and social support within this population, and the results contradicted the traditional commonsense solutions.

First of all, the results of this study showed that the average EQ-5D index score for the Chinese rural elderly was (0.78 ± 0.16). This might be related to the fact that the Chinese government has paid high-level attention to improving the basic medical and health service system, raising the social pensions of rural residents, and implementing precision poverty alleviation in recent years.

Not surprisingly, this study indicated that both formal and informal social support could generally improve HRQOL for the elderly, generally confirming the findings of previous studies [6,7,8,9,10,11,15]. The social support provided by families, communities and governments together forms the social support system of the Chinese rural elderly. Simultaneously, emotional and instrumental support from family were still important to the elderly in rural China, although the traditional function of informal care provided by family members and their networks had been weakened due to the one child policy and geographical mobility [41]. Social network support is provided by the community, while the social welfare system is provided by government. This indicates that there is a certain degree of complementarity between formal social support and informal social support. The former can compensate for the latter economically, but psychological comfort and emotional support from family members cannot be replaced. The results of this study are consistent with previous studies [16,17,18,19,20,21,22,23,24,25,26,27,28].

More importantly, our findings confirmed that the quality of social support may have a greater impact on the HRQOL of the Chinese rural elderly than the quantity of individuals in one’s social network. With respect to formal social support, the number of social activities, the frequency of participation in social activities, and the size of the pension insurance received by the elderly each month were highly positively correlated with HRQOL. Similarly, with respect to informal social support, the number of children, the frequency of contact with children, and economic support from family were also positively correlated with the HRQOL. In contrast, the quantitative variables of total social support were not statistically significant. These results were also consistent with the findings reported in previous studies [24,25,26,27,28,29,30,31,32].

It is worth noting that the traditional belief of “more children, more blessings” has begun to undergo profound changes in rural areas in recent years. Most previous research considered that family support received by the elderly in terms of living, care and emotional comfort was the most important factor [34,35,36,37,38,39,40,41,42,43,44]. However, the results of our study indicate that increased numbers of children do not contribute to higher EQ-5D index scores for the rural elderly. This may be due to the fact that with the spread of urbanization, a large number of young people have moved from rural to urban areas. The traditional family structure, values, and intergenerational relationships have undergone profound changes, and the family pension function also faces great challenges [33,35,36,37]. 

The strength of this study was that the data were obtained from CHARLS, which is a nationwide population survey with a very high response rate. Therefore, we were able to draw general conclusions, and the results can be considered representative of the whole population of the Chinese rural elders. Furthermore, most previous studies have used the TTO utility value integration system [42] to calculate the quality of life utility value for the elderly in China. The differences in population characteristics were carefully considered in this study. However, some limitations of this study should be considered. First, because our study was a cross-sectional survey, no causal relationships can be derived with respect to the association between social support and HRQOL. Longitudinal studies are needed to confirm the effects of social support on HRQOL. Second, as reported, there might exist a ceiling effect associated with measuring health status using the EQ-5D [43,44]. Third, the samples in this study could be extended in order to validate the conclusion, and refined in order to explore the comparison between different areas in China.

## 5. Conclusions

In summary, the results of the study demonstrate that the EQ-5D index scores of the elderly in rural China are above the median level, and some factors of formal and informal social support are positively related to HRQOL among the Chinese rural elders. With respect to formal social support, medical or pension insurance and social activities contribute to better life quality, and with respect to informal social support, the traditional belief that ‘the more children, the more blessings’ should be changed in future. Our results indicate that much more attention should be paid to those among the rural elderly who do not have any informal social support, and appropriate policies aiming to improving the quality of formal social support should be prioritized as targeted interventions. These results are expected to be significant for accurately improving the quality of life of the Chinese rural elderly with respect to social support.

## Figures and Tables

**Table 1:** The relationship between EQ-5D index scores and sociodemographic characteristic variables.

Variables	*n* (%)/M ± SD	EQ-5D Index Scores	Z/H/rs^1^	*P* Value
Total Number	4189	0.78 ± 0.16	-	-
Age (year)	68.40 ± 6.66	-	-	-
Gender				
Male	2450 (58.49)	0.81 ± 0.01	−16.78	<0.001
Female	1739 (41.51)	0.74 ± 0.01		
Education level				
Illiterate	1053 (25.14)	0.72 ± 0.17	0.27	<0.001
Primary school	2211 (52.78)	0.79 ± 0.15		
Middle school	631 (15.06)	0.83 ± 0.13		
High school and above	294 (7.02)	0.85 ± 0.12		
Marital status				
Married	2996 (71.52)	0.80 ± 0.15	−10.44	<0.001
Others ^a^	1193 (28.48)	0.74 ± 0.17		
Living style				
Living alone	2718 (64.88)	0.78 ± 0.01	−1.17	0.24
Living with others	1471 (35.12)	0.79 ± 0.01		
Number of chronic diseases				
None	1068 (25.50)	0.83 ± 0.13	84.33	<0.001
1type	1099 (26.24)	0.80 ± 0.15		
2–3 types	1469 (35.07)	0.76 ± 0.16		
4 types or more	553 (13.20)	0.70 ± 0.18		
Disability status				
Not disabled	2965 (70.78)	0.80 ± 0.03	12.17	<0.001
Disabled	1224 (29.22)	0.74 ± 0.05		

^a^ Unmarried/ Divorced/ Widowed. ^1^ The Wilcoxon rank-sum test, Kruskal-Wallis test and Spearman rank correlation analysis were used to examine the differences between groups in these variables, and the test statistics were expressed as Z, H and rs, respectively.

**Table 2:** The relationships between HRQOL and the quantity and quality of formal social support.

Variables	*n* (%)/M ± SD	EQ-5D Index Scores	Z/H/rs^1^	*P* Value
Number of social activities	0.78 ± 0.87	-	0.12	<0.001
Number of types of medical insurance	0.85 ± 0.48	-	0.07	<0.001
Urban and rural residents medical insurance				
Yes	932 (22.25)	0.79 ± 0.15	−3.02	0.003
No	3257 (77.75)	0.77 ± 0.16		
Government medical insurance				
Yes	105 (2.51)	0.85 ± 0.12	−5.48	<0.001
No	4084 (97.49)	0.78 ± 0.16		
Private medical insurance				
Yes	37 (0.88)	0.80 ± 0.13	−0.26	0.799
No	4152 (99.12)	0.78 ± 0.16		
Medical aid				
Yes	37 (0.88)	0.75 ± 0.20	0.58	0.564
No	4152 (99.12)	0.78 ± 0.16		
Other medical insurance				
Yes	137 (3.27)	0.80 ± 0.13	0.48	0.633
No	4052 (96.73)	0.78 ± 0.16		
Number of types of pension insurance	0.88±0.52	-	0.02	0.22
Employee pension insurance				
Yes	305 (7.28)	0.86 ± 0.11	−10.18	<0.001
No	3882 (92.72)	0.78 ± 0.16		
Urban and rural residents pension insurance				
Yes	2720 (64.96)	0.78 ± 0.16	5.16	<0.001
No	1467 (35.04)	0.80 ± 0.16		
Old age pension allowance				
Yes	430 (10.27)	0.76 ± 0.17	2.65	0.008
No	3757 (89.73)	0.78 ± 0.15		
Commercial pension insurance				
Yes	151 (3.61)	0.83 ± 0.13	−4.67	<0.001
No	4036 (96.39)	0.78 ± 0.16		
Frequency of participating in social activities				
Never	2730 (65.17)	0.77 ± 0.16	0.08	<0.001
Sometimes	407 (9.72)	0.80 ± 0.14		
Always	1052 (25.11)	0.80 ± 0.14		
Amount of pension insurance received monthly (ln)	4.86 ± 1.24	-	0.21	<0.001

^1^ The Wilcoxon rank-sum test, Kruskal-Wallis test and Spearman rank correlation analysis were used to examine the differences between groups in these variables, and the test statistics were expressed as Z, H and rs, respectively.

**Table 3:** The relationships between HRQOL and the quantity and quality of informal social support.

Variables	*n* (%)/M ± SD	EQ-5D Index Scores	Z/H/rs^1^	*P* value
Spouses				
Yes	2742 (65.46)	0.80 ± 0.15	−9.97	<0.001
No	1447 (34.54)	0.75 ± 0.17		
Number of children	3.28 ± 1.56	-	−0.15	<0.001
Number of grandchildren	0.54 ± 0.79	-	0.06	<0.001
Frequency of meeting with children				
Never	256 (6.11)	0.79 ± 0.17	0.01	0.58
Sometimes	1366 (32.61)	0.78 ± 0.15		
Always	2567 (61.28)	0.78 ± 0.16		
Frequency of contacting with children				
Never	1054 (25.16)	0.78 ± 0.17	0.04	0.01
Sometimes	772 (18.43)	0.76 ± 0.16		
Always	2363 (56.41)	0.79 ± 0.15		
Whether to take care of grandchildren				
Yes	1301 (31.06)	0.80 ± 0.15	0.05	<0.001
No	2888 (68.94)	0.78 ± 0.16		
Financial support from family(ln)	5.49 ± 1.33	-	0.14	<0.001

^1^ The Wilcoxon rank-sum test, Kruskal-Wallis test and Spearman rank correlation analysis were used to examine the differences between groups in these variables, and the test statistics were expressed as Z, H and rs, respectively.

**Table 4:** Tobit regression analyses of EQ-5D index scores and all social support variables and characteristics variables.

Variables	Model 1		Model 2		Model 3		Model 4		Model 5	
	*β*	SE	*β*	SE	*β*	SE	*β*	SE	*β*	SE
Age	−0.002 ***	0.001	−0.002 ***	0.001	−0.002 ***	0.001	−0.002 **	0.001	−0.001 *	0.001
Gender (Ref: Female)										
Male	0.045 ***	0.005	0.046 ***	0.005	0.045 ***	0.006	0.043 ***	0.006	0.046 ***	0.006
Education (Ref: Illiterate)										
Primary school	0.039 ***	0.006	0.034 ***	0.006	0.034 ***	0.007	0.033 ***	0.007	0.033 ***	0.007
Middle school	0.063 ***	0.008	0.050 ***	0.008	0.052 ***	0.009	0.052 ***	0.009	0.053 ***	0.01
High school and above	0.087 ***	0.01	0.057 ***	0.01	0.051 ***	0.013	0.051 ***	0.013	0.047 ***	0.013
Marital status (Ref: Unmarried/Divorced/Widowed)										
Married	−0.015 **	0.005	0.013 *	0.005	0.009	0.006	−0.001	0.012	−0.007	0.013
Living style (Ref: Living with others)										
Living alone	0.006	0.005	0.006	0.005	0.009	0.005	0.009	0.006	0.01	0.006
Number of chronic diseases (Ref: None)										
One type	−0.021 ***	0.006	−0.021 **	0.006	−0.016 **	0.007	−0.016 *	0.007	−0.023 **	0.008
Two to three types	−0.056 ***	0.006	−0.056 ***	0.006	−0.052 ***	0.007	−0.052 ***	0.007	−0.058 ***	0.008
Four types or more	−0.118 ***	0.008	−0.120 ***	0.007	−0.117 ***	0.009	−0.116 ***	0.009	−0.122 ***	0.009
Disability status (Ref: Not disabled)										
Disabled	−0.037 ***	0.005	−0.037 ***	0.005	−0.036 ***	0.006	−0.036 ***	0.006	−0.030 ***	0.006
Amount of medical insurance			−0.022 *	0.01	−0.020	0.012	−0.020	0.012	−0.020	0.013
Amount of pension insurance			0.025	0.015	−0.023	0.018	−0.024	0.018	−0.016	0.02
Number of social activities			0.016 ***	0.002	0.012 ***	0.004	0.012 **	0.004	0.009 *	0.004
Urban and rural residents’ medical insurance (Ref: Not participate in urban and rural residents medical insurance)										
Yes			0.032 **	0.011	0.026	0.014	0.027	0.014	0.026	0.015
Government medical insurance (Ref: No government medical insurance)										
Yes			0.047 **	0.018	0.034	0.023	0.036	0.023	0.026	0.025
Employee pension insurance (Ref: No employee pension insurance)										
Yes			0.017	0.018	−0.006	0.019	−0.005	0.019	−0.033	0.021
Urban and rural residents’ pension insurance (Ref: No urban and rural residents pension insurance)										
Yes			−0.034 *	0.016	0.016	0.018	0.017	0.018	−0.003	0.02
Old age pension allowance (Ref: No old age pension allowance)										
Yes			−0.023	0.017	0.02	0.019	0.021	0.019	0.005	0.02
Commercial pension insurance (Ref: No commercial pension insurance)										
Yes			−0.003	0.019	0.036	0.022	0.036	0.022	0.024	0.024
Frequency of participating in social activities (Ref: Never participating in social activities)										
Sometimes					0.008	0.01	0.009	0.01	0.009	0.01
Always					0.017 *	0.008	0.017 *	0.008	0.016 *	0.008
Amount of pension insurance received monthly (ln)					0.023 ***	0.004	0.022 ***	0.004	0.024 ***	0.004
Whether having spouse (Ref: No spouse)										
Yes							0.011	0.006	0.009	0.012
Number of children							−0.005 **	0.002	−0.009 ***	0.002
Number of grandchildren							−0.002	0.003	−0.007	0.005
Frequency of contacting with children (Ref: Never contacting with children)										
Sometimes									0.002	0.009
Always									0.012*	0.007
Whether to take care of grandchildren (Ref: Do not take care of grandchildren)										
Yes									0.008	0.008
Financial support from family (ln)									0.011 ***	0.002

* *P* < 0.05; ** *P* < 0.01; *** *P* < 0.001.
